# Differential impacts of clinical, anatomical, and procedural factors on early and late mortality following open thoracoabdominal aortic repair: a retrospective observational study

**DOI:** 10.1186/s13019-024-02933-2

**Published:** 2024-06-24

**Authors:** Jin Kyoung Kim, Gwan Sic Kim, Woo Seok Kim, Ho Jin Kim, Suk Jung Choo, Joon Bum Kim

**Affiliations:** 1 Department of Thoracic and Cardiovascular Surgery, Asan Medical Center, University of Ulsan College of Medicine, 88, Olympic-ro 43-gil, Songpa-gu, Seoul, 138-736, 05505 Republic of Korea; 2 Department of Thoracic and Cardiovascular Surgery, Ulsan University Hospital, University of Ulsan College of Medicine, Ulsan, Republic of Korea

**Keywords:** Thoracoabdominal aortic aneurysm, Surgery, Mortality, Complication, Risk factor

## Abstract

**Background:**

The operative outcomes of thoracoabdominal aortic aneurysms (TAAAs) are challenged by high operative mortality and disabling complications. This study aimed to explore the baseline clinical, anatomical, and procedural risk factors that impact early and late outcomes following open repair of TAAAs.

**Methods:**

We reviewed the medical records of 290 patients who underwent open repair of TAAAs between 1992 and 2020 at a tertiary referral center. Determinants of early mortality (within 30 days or in hospital) were analyzed using multivariable logistic regression models, while those of overall follow-up mortality were explored using multivariable Cox proportional hazards models and landmark analyses.

**Results:**

The rates of early mortality and spinal cord deficits were 13.1% and 11.0%, respectively, with Crawford extent II showing the highest rates. In the logistic regression models, older age (*P* < 0.001), high cardiopulmonary bypass (CPB) time (*P* < 0.001), and low surgical volume of the surgeon (*P* < 0.001) emerged as independent factors significantly associated with early mortality. During follow-up (median, 5.0 years; interquartile range, 1.1–7.6 years), 82 late deaths occurred (5.7%/patient-year). Cox proportional hazards models demonstrated that older age (*P* < 0.001) and low hemoglobin level (*P* = 0.032) were significant risk factors of overall mortality, while the landmark analyses revealed that the significant impacts of low surgical volume (*P* = 0.017), high CPB time (*P* = 0.002), and Crawford extent II (*P* = 0.017) on mortality only remained in the early postoperative period, without significant late impacts (all *P* > 0.05).

**Conclusion:**

There were differential temporal impacts of perioperative risk variables on mortality in open repair of TAAAs, with older age and low hemoglobin level having significant impacts throughout the postoperative period, and low surgical volume, high CPB time, and Crawford extent II having impacts in the early postoperative phase.

**Supplementary Information:**

The online version contains supplementary material available at 10.1186/s13019-024-02933-2.

## Background

Despite the development of endovascular therapies for the management of aortic diseases, open surgical repair remains the gold standard approach for the treatment of thoracoabdominal aortic aneurysms (TAAAs) [1]. This is because of the limited efficacy of currently available endovascular devices for the thoracoabdominal aorta. The complexity of aortic branching vessels, such as visceral, renal, and spinal cord feeding arteries, hinders effective exclusion of the diseased aorta by endovascular stenting. However, the operative outcomes of TAAAs are still challenged by high rates of operative mortality and disabling complications [2]. As most available data on the operative results of open repair of TAAAs have been obtained by groups of leading experts, they have limited value for estimating the operative outcomes of TAAAs in real-world practice [2, 3]. Moreover, limited studies have comprehensively explored the underlying risk factors contributing to adverse outcomes with consideration of the potential differential temporal impacts of such variables on long-term survival outcomes in open repair of TAAAs. In order to contribute to the body of evidence on this issue, we aimed to describe the outcomes of open repair of TAAAs and identify the predictors of adverse operative outcomes by analyzing long-term follow-up data.

## Methods

### Patients

We identified 290 consecutive adult patients who underwent TAAA repair between September 1992 and December 2020, from the institutional database of Asan Medical Center, Seoul, Korea. The extent of TAAA repair was categorized according to the Crawford extent classification [4]. Early death was defined as death occurring within 30 days of surgery or before discharge. A disabling complication, which persisted beyond the index hospitalization or up to early mortality, was defined as a composite of a disabling neurologic injury and disabling end-organ failure, such as a condition requiring bowel resection or permanent dialysis, that occurred postoperatively. Neurologic deficits (both cerebral and spinal) were categorized as disabling and nondisabling, with the latter being completely resolved without any residual neurologic symptoms within the index hospitalization. Low cardiac output syndrome (LOCS) was defined as hemodynamic compromise requiring mechanical support, such as extracorporeal membrane oxygenation, an intra-aortic balloon pump, or a ventricular assist device, postoperatively. The study protocol was approved by the institutional review board of Asan Medical Center (2021 − 0657), which waived the requirement for informed consent from individual patients owing to the retrospective nature of the study.

### Surgical technique

The standard approach included double-lumen endotracheal intubation; a thoracoabdominal incision via the fourth, fifth, or sixth intercostal spaces depending on the target aortic lesions; a retroperitoneal approach to access the abdominal aorta; and a circumferential incision of the diaphragm. The left femoral artery and vein were exposed through a groin incision, and the sequential aortic clamping strategy was applied whenever possible to minimize the segmental artery ischemic time. In cases where proximal clamping was deemed to not be feasible (i.e., concomitant distal arch repair), deep hypothermic circulatory arrest was used, during which body cooling was aimed at a nasopharyngeal temperature of 16℃ to 28℃ depending on the surgeon’s preferences. Otherwise, partial cardiopulmonary bypass (CPB) was the main method to establish lower body circulation during aortic clamping. When aortic replacement involved visceral/renal segments (i.e., Crawford extent II, III, or IV [some cases]), visceral perfusion with tepid blood and renal perfusion with tepid blood or intermittent cold crystalloid were performed using separate balloon cannulae. Throughout the study period, there were variations in the manner of reattaching segmental arteries (island patch vs. individual grafting) and the adoption of preoperative cerebrospinal fluid drainage and intraoperative neurophysiologic monitoring depending on preferences among different attending surgeons.

### Statistical analysis

Categorical variables are presented as numbers and percentages, and continuous variables are presented as mean ± standard deviation or median with range, as appropriate. For multivariable risk factor analyses of early mortality and the composite of early mortality and disabling complications, logistic regression models were used. All baseline and procedural variables were evaluated in univariable logistic regression models, and those with a *P*-value ≤ 0.10 in the univariable analyses were included in the multivariable logistic regression models. Multivariable analyses involved a stepwise backward elimination technique, and only variables with a *P*-value < 0.10 were included in the final model. The multicollinearity in the multivariable regression model was tested by examining the variance inflation factor (VIF) for all variables retained in the final model, and covariates with values ≥ 2.5 were considered as having considerable collinearity [5]. Among the significant risk factors of early mortality determined by the final logistic regression model, continuous variables were also assessed by receiver operating characteristic (ROC) curve analysis to determine the area under the curve (AUC) and the best cutoff values showing the greatest accuracy (sensitivity + specificity) to differentiate mortality status.

Kaplan–Meier curves were constructed to delineate overall survival and freedom from additional aortic intervention, and the log-rank test was performed to compare intergroup differences in the estimates. Cox proportional hazards models were used to evaluate the hazard ratio (HR) of overall mortality throughout the follow-up for each risk covariate, and stepwise multivariable modeling was used to identify independent risk factors. For this, the Cox proportional hazards assumption was examined by using log (− log[survival]) curves and testing partial (Schoenfeld) residuals. For variables showing significant violation (Schoenfeld residual *P* < 0.05), landmark mortality analyses were performed with a split into 2 intervals at appropriate time points, in which proportional hazards assumptions were further tested within the given time frames [6].

A total of 8 surgeons were involved in this study. Of these surgeons, 3 had a surgical volume of > 70 cases and 5 had a surgical volume of < 20 cases. We merged the outcomes of low-volume surgeons (< 20 cases) into a single category to investigate the impact of low surgical volume on study outcomes. A *P*-value < 0.05 was considered statistically significant. R statistical software (version 4.0.5; R Project for Statistical Computing, Vienna, Austria) was used for statistical analyses.

## Results

### Baseline and operative characteristics

The baseline variables are summarized in Table 1. Of the 290 patients, 87 (30.0%) were female, and the mean patient age was 56.5 ± 14.3 years. The most common Crawford extent was extent II (*n* = 106, 36.6%), followed by extent I (*n* = 93, 32.1%). The underlying aortic pathology was chronic dissection in 150 (51.7%) patients, acute or subacute dissection in 25 (8.6%) patients, and chronic aneurysm without dissection in 110 (37.9%) patients. Half of the patients (*n* = 145) had prior aortic repairs, and 12 (4.1%) patients underwent redo-thoracotomy. The mean maximum aortic diameter was 64.2 ± 16.9 mm. Emergent or urgent operations were performed in 60 (20.7%) patients with aortic dissection or aortic rupture. A cerebrospinal fluid drain was placed preoperatively in 182 (62.8%) patients. The mean CPB time was 155.4 ± 93.4 min. Total circulatory arrest (TCA) was used in 96 (33.1%) patients, and the mean TCA time was 19.2 ± 17.1 min. The intercostal or lumbar arteries were reattached in 105 (36.2%) patients. The operative details are summarized in Table 2.

**Table 1:** Preoperative characteristics according to crawford extent

	Total	Extent I	Extent II	Extent III	Extent IV	Extent V	
Variable	*n* = 290	*n* = 93 (32.1)	*n* = 106 (36.6)	*n* = 63 (21.7)	*n* = 8 (2.8)	*n* = 20 (6.9)	*P* value
Age (years), mean ± SD	56.5 ± 14.3	61.6 ± 11.5	50.8 ± 14.5	56.2 ± 14.0	50.8 ± 22.0	65.7 ± 8.0	< 0.001
Female sex, n (%)	87 (30.0)	31 (33.3)	32 (30.2)	13 (20.6)	2 (5.0)	9 (45.0)	0.25
BMI, mean ± SD	23.8 ± 4.0	24.5 ± 3.9	23.8 ± 4.0	23.1 ± 4.0	20.9 ± 4.2	23.5 ± 3.4	0.06
Hypertension, n (%)	205 (70.7)	71 (76.3)	75 (70.8)	36 (57.1)	7 (87.5)	16 (80.0)	0.06
Dyslipidemia, n (%)	62 (21.4)	27 (29.0)	19 (17.9)	8 (12.7)	0 (0.0)	8 (40.0)	0.011
Diabetes mellitus, n (%)	28 (9.7)	12 (12.9)	10 (9.4)	4 (6.3)	0 (0.0)	2 (10.0)	0.60
Aorta pathology							
Rupture or impending rupture, n (%)	31 (10.7)	8 (8.6)	8 (7.5)	9 (14.3)	0 (0.0)	6 (30.0)	0.025
Chronic dissection, n (%)	150 (51.7)	47 (50.5)	75 (70.8)	21 (33.3)	5 (62.5)	2 (10.0)	< 0.001
Acute or Subacute dissection, n (%)	25 (8.6)	10 (10.8)	8 (7.5)	5 (7.9)	0 (0.0)	2 (10.0)	0.82
Aneurysm without dissection, n (%)	110 (37.9)	33 (35.5)	22 (20.8)	37 (58.7)	3 ( 37.5)	15 (75.0)	< 0.001
Previous aortic intervention							
Prior aortic repair, n (%)	145 (50.0)	42 (45.2)	59 (55.7)	33 (52.4)	5 (62.5)	6 (30.0)	0.19
Previous TEVAR, n (%)	11 (3.8)	6 (6.5)	1 (0.9)	2 (3.2)	0 (0.0)	2 (10.0)	0.16
Previous PCI, n (%)	19 (6.6)	4 (4.3)	8 (7.5)	4 (6.3)	0 (0.0)	3 (15.0)	0.43
Previous CABG, n (%)	11 (3.8)	7 (7.5)	1 (0.9)	3 (4.8)	0 (0.0)	0 (0.0)	0.13
Prior myocardial infarction, n (%)	1 (0.3)	1 (1.1)	0 (0.0)	0 (0.0)	0 (0.0)	0 (0.0)	0.71
Prior CVA or TIA, n (%)	14 (4.8)	2 (2.2)	7 (6.6)	4 (6.3)	0 (0.0)	1 (5.0)	0.57
Marfan syndrome, n (%)	49 (16.9)	5 (5.4)	30 (28.3)	10 (15.9)	4 (50.0)	0 (0.0)	< 0.001
Peripheral vascular disease, n (%)	6 (2.1)	3 (3.2)	1 (0.9)	2 (3.2)	0 (0.0)	0 (0.0)	0.69
Current tobacco use, n (%)	73 (25.2)	21 (22.6)	21 (19.8)	26 (41.3)	1 (12.5)	4 (20.0)	0.021
ESRD (on dialysis), n (%)	18 (6.2)	5 (5.4)	5 (4.7)	5 (7.9)	1 (12.5)	2 (10.0)	0.76
Chronic lung disease, n (%)	16 (5.5)	7 (7.5)	4 (3.8)	2 (3.2)	0 (0.0)	3 (15.0)	0.21
GFR (mL/min/1.73 m^2^), mean ± SD	75.9 ± 32.8	72.5 ± 27.6	78.1 ± 31.4	78.8 ± 39.7	71.9 ± 40.5	72.1 ± 35.8	0.68
Blood hemoglobin level (g/dL), mean ± SD	12.5 ± 1.8	12.7 ± 2.0	12.6 ± 1.7	12.2 ± 1.7	12.1 ± 2.0	12.1 ± 1.8	0.42
Symptomatic, n (%)	126 (43.4)	37 (39.8)	49 (46.2)	30 (47.6)	3 (37.5)	7 (35.0)	0.74
Maximum aortic diameter (mm), mean ± SD	64.2 ± 16.9	65.4 ± 19.0	63.2 ± 16.8	65.6 ± 13.0	58.0 ± 14.7	62.2 ± 19.1	0.64
Operator							0.005
1	91 (31.4)	28 (30.1)	34 (32.1)	12 (19.0)	3 (37.5)	14 (70.0)	
2	72 (24.8)	19 (20.4)	32 (30.2)	21 (33.3)	0 (0.0)	0 (0.0)	
3	87 (30.0)	34 (36.6)	26 (24.5)	19 (30.2)	4 (50.0)	4 (20.0)	
4*	40 (13.8)	12 (12.9)	14 (13.2)	11 (17.5)	1 (12.5)	2 (10.0)	

Data are presented as n (%) or mean ± SD.

BMI, body mass index; TEVAR, thoracic endovascular aneurysm repair; PCI, percutaneous coronary intervention; CABG, coronary artery bypass grafting; CVA, cerebrovascular accident; TIA, transient ischemic attack; ESRD, end-stage renal disease; GFR, glomerular filtration rate

*Operator 4 refers to a group of five less experienced operators who had case volumes of less than 20 (range, 1–18)

**Table 2:** Operative details according to crawford extent

	Total	Extent I	Extent II	Extent III	Extent IV	Extent V	
Variable	*n* = 290	*n* = 93 (32.1)	*n* = 106 (36.6)	*n* = 63 (21.7)	*n* = 8 (2.8)	*n* = 20 (6.9)	*P* value
Urgent or emergent surgery, n (%)	60 (20.7)	19 (20.4)	19(17.9)	14 (22.2)	1 (12.5)	7 (35.0)	0.49
CPB time (min), mean ± SD	155.4 ± 93.4	145.1 ± 73.9	239.8 ± 107.4	176.5 ± 114.0	128.5 ± 93.4	87.2 ± 78.3	< 0.001
Total circulatory arrest, n (%)	96 (33.1)	28 (32.6)	56 (54.4)	10 (18.2)	0 (0.0)	2 (10.0)	< 0.001
Total circulatory arrest time (min), mean ± SD	19.2 ± 17.1	19.1 ± 11.2	23.8 ± 25.9	28.4 ± 29.3	0.0 ± 0.0	5.5 ± 2.1	0.47
Aortic repair details							
Redo-thoracotomy, n (%)	12 (4.1)	0 (0.0)	3 (2.8)	6(9.5)	2 (25.0)	1 (5.0)	0.001
Clamping proximal to the LSCA, n (%)	19 (6.6)	11 (12.0)	8 (7.5)	0 (0.0)	0 (0.0)	0 (0.0)	0.028
Extraction of TEVAR, n (%)	8 (2.8)	5 (5.4)	0 (0.0)	2 (3.2)	0 (0.0)	1 (5.0)	0.20
Reverse elephant trunk, n (%)	13 (4.5)	5 (5.4)	8 (7.5)	0 (0.0)	0 (0.0)	0 (0.0)	0.15
Completion of elephant trunk, n (%)	7 (2.4)	3 (3.2)	4 (3.8)	0 (0.0)	0 (0.0)	0 ( 0.0)	0.50
ICA/LA reattachment, n (%)	105 (36.2)	28 (30.1)	57 (53.8)	14 (22.2)	2 (25.0)	4 (20.0)	< 0.001
Bi-iliac artery reconstruction, n (%)	36 (12.4)	1 (1.1)	19 (17.9)	16 (25.4)	0 (0.0)	0 (0.0)	< 0.001
Splenectomy, n (%)	6 (2.1)	2 (2.2)	2 (1.9)	2 (3.2)	0 (0.0)	0 (0.0)	0.91
Operative adjuncts							
Cerebrospinal fluid drainage, n (%)	182 (62.8)	57 (61.3)	74 (69.8)	40 (63.5)	6 (75.0)	5 (25.0)	0.005

Data are presented as n (%) or mean ± SD.

CPB, cardiopulmonary bypass; TCA, total circulatory arrest; LSCA, left subclavian artery; TEVAR, thoracic endovascular aneurysm repair; ICA, intercostal artery; LA, lumbar artery

### Early outcomes

The rate of early mortality in the overall study population was 13.1% (*n* = 38; Table 3), and the rate was the highest for extent II (19.8%), followed by extent III (12.7%), extent V (10.0%), and extent I (7.5%). There was no case of early mortality involving extent IV. Disabling complications occurred in 48 (16.6%) patients, including disabling cerebral injuries in 23 (7.9%), permanent spinal cord injuries in 23 (7.9%), and bowel resection in 8 (2.8%). Overall, the composite rate of operative morality and disabling complications was 24.1% (*n* = 70). Details of early postoperative adverse events according to Crawford extent classification are summarized in Table 3.

**Table 3:** Early outcomes according to crawford extent

	Total	Extent I	Extent II	Extent III	Extent IV	Extent V	
Variable	*n* = 290	*n* = 93 (32.1)	*n* = 106 (36.6)	*n* = 63 (21.7)	*n* = 8 (2.8)	*n* = 20 (6.9)	*P* value
Operative mortality, n (%)	38 (13.1)	7 (7.5)	21 (19.8)	8 (12.7)	0 (0.0)	2 (10.0)	0.09
Cerebral complication (composite), n (%)	42 (14.5)	16 (17.2)	18 (17.0)	6 (9.5)	2 (25.0)	0 (0.0)	0.17
Intracranial hemorrhage, n (%)	8 (2.8)	2 (2.2)	2 (1.9)	4 (4.8)	0 (0.0)	0 (0.0)	0.74
Seizure, n (%)	13 (4.5)	8 (7.5)	5 (5.4)	0 (0.0)	0 (0.0)	0 (0.0)	0.15
Nondisabling stroke, n (%)	1 (0.3)	1 (1.1)	0 (0.0)	0 (0.0)	0 (0.0)	0 (0.0)	0.71
Disabling stroke, n (%)	20 (6.9)	5 (4.7)	11 (11.8)	2 (3.2)	2 (25.0)	0 (0.0)	0.024
Spinal cord deficit (composite), n (%)	32 (11.0)	6 (6.5)	18 (17.0)	7 ( 11.1)	1 (12.5)	0 (0.0)	0.08
Permanent SCD, n (%)	23 (7.9)	6 (6.5)	13 (12.3)	4 (6.3)	0 (0.0)	0 (0.0)	0.23
Permanent paraplegia, n (%)	19 (7.0)	4 (5.4)	11 (9.4)	4 (6.3)	0 (0.0)	0 (0.0)	0.46
Permanent paraparesis, n (%)	4 (1.4)	2 (2.2)	2 (1.9)	0 (0.0)	0 (0.0)	0 (0.0)	0.76
Temporary SCD, n (%)	9 (3.1)	0 (0.0)	5 (4.7)	3 (4.8)	1 (1.3)	0 (0.0)	0.11
Temporary paraplegia, n (%)	6 (2.0)	0 (0.0)	4 (3.8)	2 (3.2)	0 (0.0)	0 (0.0)	0.35
Temporary paraparesis, n (%)	3 (1.0)	0 (0.0)	1 (0.9)	1 (1.6)	1 (12.5)	0 (0.0)	0.020
Gastrointestinal complication (composite), n (%)	12 (4.1)	2 (2.2)	3 (2.8)	6 (9.5)	0 (0.0)	1 (5.0)	0.17
Gastrointestinal ischemia, n (%)	4 (1.0)	0 (0.0)	1 (0.9)	3 (4.8)	0 (0.0)	0 (0.0)	0.92
Gastrointestinal resection, n (%)	8 (2.8)	2 (2.2)	2 (1.9)	3 (4.8)	0 (0.0)	1 (5.0)	0.74
Bleeding requiring reoperation, n (%)	27 (9.3)	4 (4.3)	18 (17.0)	3 (4.8)	0 (0.0)	2 (10.0)	0.014
New dialysis, n (%)	54 (18.6)	15 (16.1)	28 (26.4)	10 (15.9)	0 (0.0)	1 (5.0)	0.06
Low cardiac output syndrome, n (%)	25 (8.6)	3 (3.2)	16 (15.1)	4 (6.3)	0 (0.0)	2 (10.0)	0.036
Multiple organ failure, n (%)	17 (5.9)	2 (2.2)	11 (10.4)	3 (4.8)	0 (0.0)	1 (5.0)	0.14
Surgical wound revision, n (%)	30 (10.3)	5 (5.4)	16 (15.1)	7 (11.1)	1 (12.5)	1 (5.0)	0.22
Pneumonia, n (%)	51 (17.6)	16 (17.2)	23 (21.7)	9 (14.3)	1 (12.5)	2 (10.0)	0.62
Drainage of pericardial effusion, n (%)	14 (4.8)	2 (2.2)	10 (9.4)	2 (3.2)	0 (0.0)	0 (0.0)	0.09
Tracheostomy, n (%)	55 (19.0)	13 (14.0)	30 (28.3)	11 (17.5)	0 (0.0)	1 (5.0)	0.017
Reintubation, n (%)	52 (17.9)	14 (15.7)	26 (28.3)	10 (18.5)	1 (12.5)	1 (5.0)	0.09
Ventilator support > 48 h, n (%)	116 (40.0)	31 (33.3)	63 (59.4)	20 (31.7)	1 (12.5)	1 (5.0)	< 0.001
Vocal cord paralysis, n (%)	34 (11.7)	19 ( 20.4)	11 ( 10.4)	4 (6.3)	0 (0.0)	0 ( 0.0)	0.014
Disabling complications, n (%)*	62 (21.4)	15 (16.1)	32 (30.2)	13 (20.6)	0 (0.0)	2 (10.0)	0.038

Data are presented as n (%)

ARF, acute renal failure; SCD, spinal cord deficit

*Disabling complications include operative mortality, disabling stroke, permanent spinal cord deficits, and gastrointestinal resection

### Multivariable risk factor analysis for early adverse events

Table 4 summarizes the findings of multivariable risk factor analysis for early mortality. In multivariable analyses, older age (odds ratio [OR], 2.12; 95% confidence interval [CI], 1.49–3.19; *P* < 0.001), low surgical volume (OR, 5.90; 95% CI, 2.10–17.01; *P* < 0.001), and high CPB time (OR, 1.13; 95% CI, 1.08–1.18; *P* < 0.001) were identified as significant independent predictors of early mortality, while blood hemoglobin level showed marginal significance (OR, 0.79; 95% CI, 0.54–0.99; *P* = 0.054). There was no violation in multicollinearity as demonstrated by VIF < 2.5 for all significant variables (Table 4). Although extent II or III emerged as a significant factor associated with early mortality, it was not retained in the final multivariable model. When extent II and the composite of extent II/III were forced into the multivariable model one by one with incorporation of significant variables, the adjusted OR was 1.67 (95% CI, 0.34–4.10; *P* = 0.257) for extent II and 1.91 (95% CI, 0.37–5.16; *P* = 0.186) for extent II/III.

**Table 4:** Univariable and final multivariable logistic regression models for early mortality

	Univariable			Multivariable			
Variable	OR	95% CI	*P* value	OR	95% CI	*P* value	VIF
**Baseline variables**							
Age (by 10-year increment)	1.57	1.21–2.08	0.001	2.12	1.49–3.19	< 0.001	1.219
Female sex	0.69	0.30–1.48	0.364				
Current smoker	1.25	0.56–2.60	0.566				
Body mass index	1.07	0.98–1.16	0.109				
Diabetes	0.90	0.25–2.48	0.850				
COPD	1.58	0.34–5.19	0.494				
Marfan syndrome	0.11	0.01–0.55	0.035				
Prior aorta repair	0.78	0.40–1.55	0.487				
Redo-thoracotomy	0.59	0.03–3.18	0.621				
GFR (by 10-mL/min/1.73 m^2^ increment)	0.81	0.74–0.90	< 0.001				
Blood hemoglobin level (by 1-g/dL increment)	0.77	0.63–0.93	0.008	0.79	0.54–0.99	0.054	1.066
**Anatomical factors**							
Maximal aortic diameter (by 1-mm increment)	1.26	1.05–1.50	0.011				
Crawford extent II (vs. others)*	2.43	1.22–4.90	0.012				
Crawford extent II or III (vs. others)**	2.58	1.22–5.98	0.019				
**Operative factors**							
Urgent/emergent surgery	1.71	0.81–3.45	0.143				
CPB time (by 10-min increment)	1.04	1.02–1.08	0.002	1.13	1.08–1.18	< 0.001	1.465
Lowest target temperature (by 1 °C increment)	0.99	0.95–1.04	0.784				
Total circulatory arrest	1.59	0.82–3.04	0.160				
Low-volume surgeons (vs. other operators)	2.64	1.13–5.85	0.020	5.90	2.10–17.00	< 0.001	1.210
Concomitant distal arch repair	2.05	0.99–4.18	0.048				

OR, odds ratio; CI, confidence interval; VIF, variance inflation factor; COPD, chronic obstructive pulmonary disease; GFR, glomerular filtration rate; CPB, cardiopulmonary bypass

*After adjusting for significant factors, Crawford extent II showed an OR of 1.67 (95% CI, 0.34–4.10; *P* = 0.257)

**After adjusting for significant factors, Crawford extent II/III showed an OR of 1.91 (95% CI, 0.37–5.16; *P* = 0.186)

For the composite outcome of early mortality and disabling complications, older age (OR, 2.07; 95% CI, 1.57–2.81; *P* < 0.001), low hemoglobin level (OR, 0.70; 95% CI, 0.58–0.85; *P* < 0.001), extent II/III (Table 5), low surgical volume (OR, 2.58; 95% CI, 1.35–4.95; *P* = 0.004), and high CPB time (OR, 1.05; 95% CI, 1.02–1.09; *P* = 0.002) were significant risk factors, while female sex was found to be protective against the composite outcomes (OR, 0.23; 95% CI, 0.10–0.49; *P* < 0.001). In these multivariable models, there was no violation in multicollinearity (VIF < 2.5; Table 5).

**Table 5:** Univariable and final multivariable logistic regression models for composite of early death and disabling complications

	Univariable			Multivariable			
Variable	OR	95% CI	*P* value	OR	95% CI	*P* value	VIF
**Baseline variables**							
Age (by 10-year increment)	1.52	1.24–1.89	< 0.001	2.07	1.57–2.81	< 0.001	1.39
Female sex	0.52	0.28–0.93	0.032	0.23	0.10–0.49	< 0.001	1.14
Current smoker	1.34	0.75–2.37	0.314				
Body mass index	1.00	0.93–1.06	0.953				
Diabetes	1.23	0.51–2.77	0.633				
COPD	1.16	0.36–3.31	0.786				
History of coronary disease	1.89	0.96–3.63	0.060				
Marfan syndrome	0.52	0.23–1.08	0.095				
Prior aorta repair	0.93	0.56–1.56	0.794				
Redo-thoracotomy	1.28	0.33–4.19	0.692				
GFR (by 10-mL/min/1.73 m^2^ increment)	0.86	0.79–0.93	< 0.001				
Blood hemoglobin level (by 1-g/dL increment)	0.72	0.62–0.84	< 0.001	0.70	0.58–0.85	< 0.001	1.14
**Anatomical factors**							
Maximal aortic diameter (by 1-mm increment)	1.17	1.00–1.36	0.045				
Crawford extent II (vs. others)	2.54	1.50–4.31	< 0.001	4.45	2.13–9.72	< 0.001	1.41
Crawford extent II or III (vs. others)	2.11	1.23–3.70	0.008	2.35	1.18–4.79	0.016	1.20
**Operative factors**							
Urgent/emergent surgery	1.98	1.08–3.58	0.025				
CPB time (by 10-min increment)	1.04	1.02–1.07	< 0.001	1.05	1.02–1.09	0.002	1.33
Lowest target temperature (by 1 °C increment)	1.00	0.96–1.04	0.861				
Total circulatory arrest	1.30	0.75–2.21	0.344				
Low-volume surgeons (vs. other operators)	2.47	1.47–4.20	< 0.001	2.58	1.35–4.95	0.004	1.05
Concomitant arch repair	1.52	0.85–2.67	0.151				

OR, odds ratio; CI, confidence interval; VIF, variance inflation factor; COPD, chronic obstructive pulmonary disease; GFR, glomerular filtration rate; CPB, cardiopulmonary bypass

For early mortality, the ROC curve yielded an AUC of 0.64 (95% CI, 0.57−0.76; *P* = 0.0023) for age, 0.66 (95% CI, 0.58−0.75; *P* = 0.0018) for hemoglobin level, 0.73 (95% CI, 0.65–0.81; *P* = 0.0016) for glomerular filtration rate (GFR), and 0.62 (95% CI, 0.53−0.71; *P* = 0.0023) for CPB time (Supplementary Fig. 1). The greatest accuracy for the prediction of mortality was obtained at the cutoff values of 64.5 years for age, 11.6 g/dL for hemoglobin level, 3.65 g/dL for albumin level, 66 mL/min/1.73 m^2^ for GFR, and 172.5 min for CPB time.

### Follow-up long-term outcomes

**Fig. 1:**
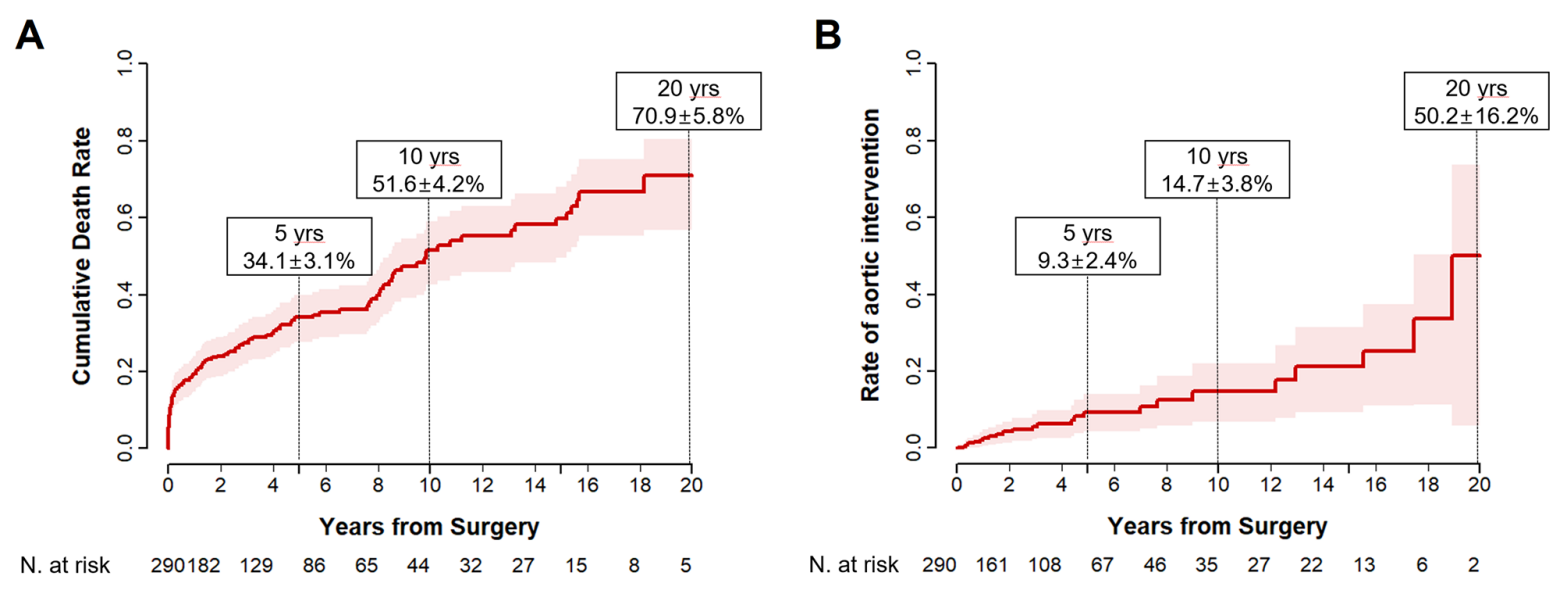
Cumulative incidence curve for mortality (**a**) and aortic reintervention (**b**) after open thoracoabdominal aortic repair

**Fig. 2:**
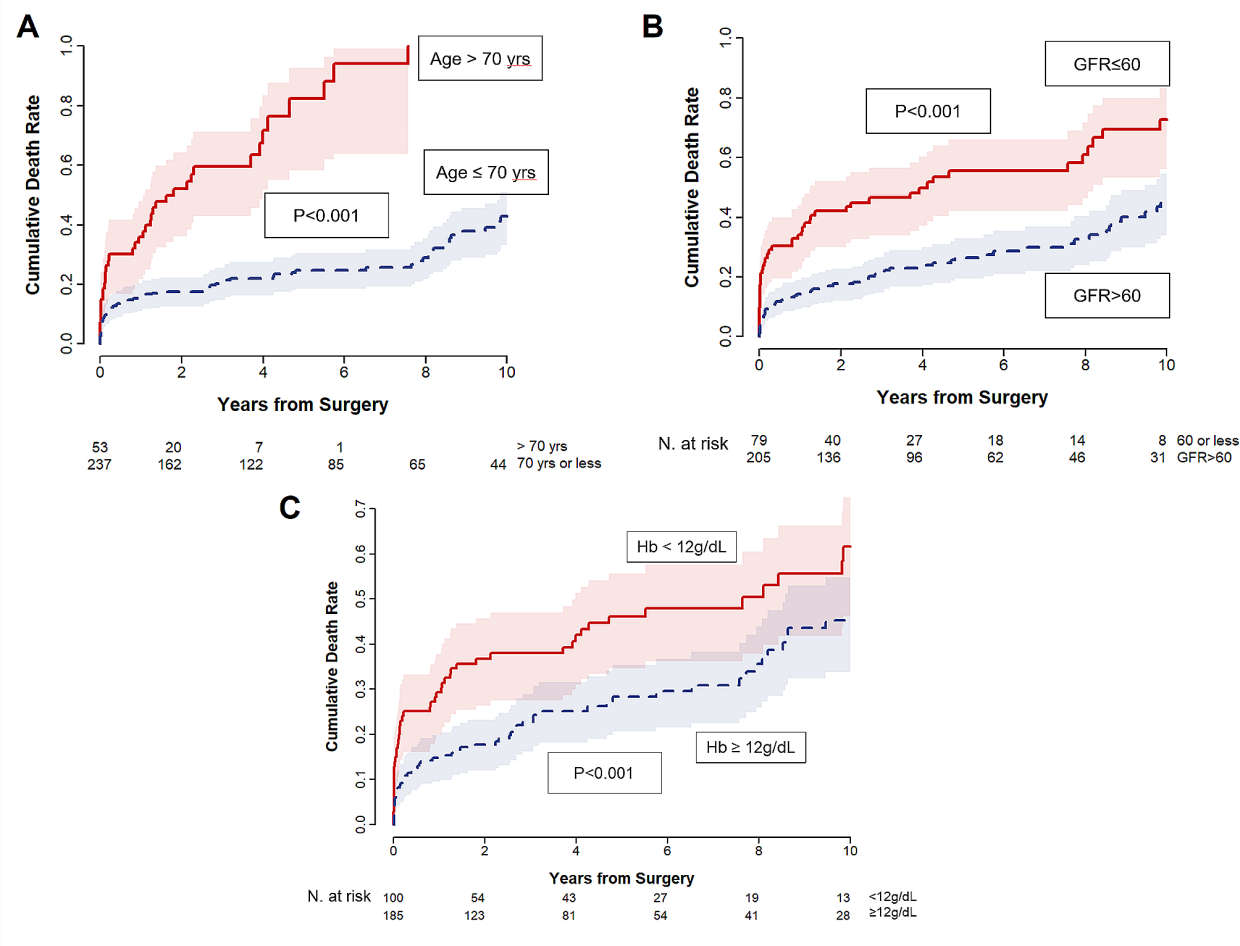
Cumulative incidence curve for mortality after open thoracoabdominal aortic repair. (**a**) Age > 70 years vs. age ≤ 70 years; (**b**) GFR ≤ 60 mL/min/1.73 m^2^ vs. GFR > 60 mL/min/1.73 m^2^; (**c**) Hb < 12 g/dL vs. Hb ≥ 12 g/dL. Hb: hemoglobin; GFR: glomerular filtration rate

**Fig. 3:**
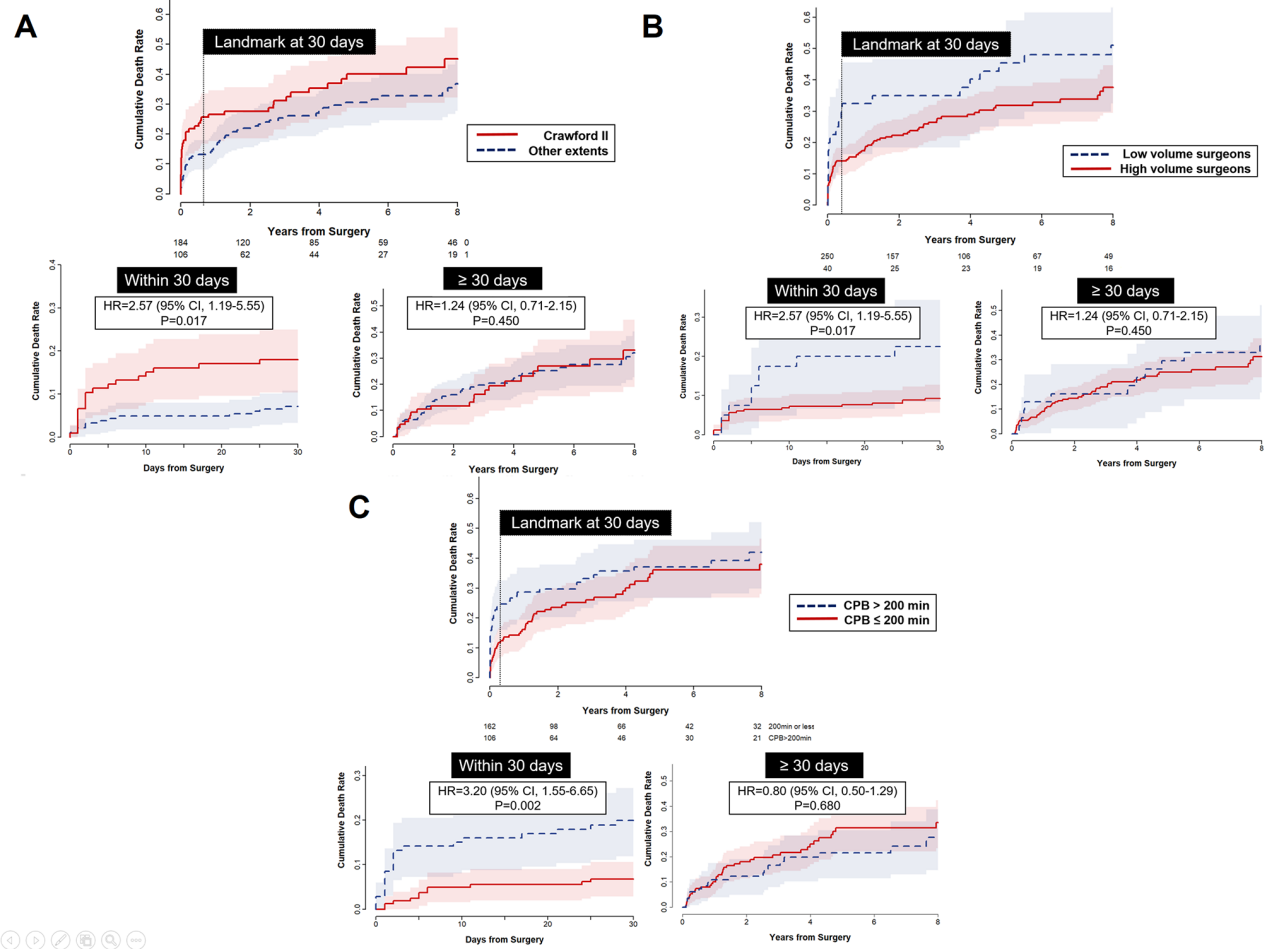
Adjusted landmark analyses for 3 variables split into 2 intervals at postoperative 30 days. (**a**) Crawford extent II vs. other extents; (**b**) low-volume surgeon vs. high-volume surgeon; (**c**) CPB > 200 min vs. CPB ≤ 200 min). CPB: cardiopulmonary bypass

During a median follow-up of 5.0 years (interquartile range, 1.1–7.6 years; overall 1440.0 patient-years), late death occurred in 82 patients. Moreover, 24 patients required aortic reintervention due to progression of the proximal (*n* = 13) or distal (*n* = 8) aortic lesions (*n* = 21), and graft infection (*n* = 3). These reinterventions were carried out by open surgical repair in all patients except one who underwent endovascular stent grafting to treat a proximal descending aortic aneurysm. The cumulative incidence rates of mortality at 5, 10, and 20 years were 34.1 ± 3.1%, 51.4 ± 4.2%, and 70.9 ± 5.8%, respectively (Fig. 1a). Moreover, the cumulative incidence rates of aortic reintervention at 5, 10, and 20 years were 9.3 ± 2.4%, 14.7 ± 3.8%, and 50.2 ± 16.2%, respectively. In the multivariable Cox proportional hazards models, age and hemoglobin level were significant factors for overall mortality, while GFR showed marginal significance (Table 6). Cumulative death rates depending on these 3 variables are illustrated in Fig. 2. Unlike these 3 variables, Crawford extent, surgical volume, and CPB time showed violation of the Cox proportional hazards assumption tested by the Schoenfeld residual (*P* < 0.05; Supplementary Fig. 2). Therefore, adjusted landmark analyses were conducted for these 3 variables with a split into 2 intervals at postoperative 30 days, and the Schoenfeld residual showed an acceptable range for these variables in the given time frames. All of these variables showed significant associations with mortality in the early phase (*P* = 0.002–0.017), while their impacts in the late phase were insignificant (*P* = 0.45–0.68) (Fig. 3).

**Table 6:** Univariable and final multivariable cox proportional hazards models for all-cause mortality

	Univariable				Multivariable		
Variable	HR	95% CI	*P* value	Schoenfeld residual *P* value	HR	95% CI	*P* value
Age (by 10-year increment)	2.04	1.69–2.45	< 0.001	0.075	1.88	1.55–2.27	< 0.001
Marfan syndrome	0.40	0.21–0.77	0.006	0.43			
GFR (by 10-mL/min/1.73 m^2^ increment)	0.88	0.83–0.92	< 0.001	0.64	0.95	0.89–1.01	0.080
Blood hemoglobin level (by 1-g/dL increment)	0.78	0.71–0.87	< 0.001	0.59	0.89	0.80–0.99	0.032
Crawford extent II (vs. others)*				0.023			
Urgent/emergent surgery	1.43	0.94–2.17	0.091	0.62			
CPB time*				< 0.001			
Low-volume surgeons (vs. other operators)*				0.041			

OR, odds ratio; CI, confidence interval; VIF, variance inflation factor; COPD, chronic obstructive pulmonary disease; GFR, glomerular filtration rate; CPB, cardiopulmonary bypass

*These variable showed significant violation in Cox proportional hazards assumption examined by the Schoenfeld residual

## Discussion

Most existing studies on open surgical repair for TAAAs have been performed by world-leading experts from high-volume centers, and the surgical techniques and strategies described in those reports have contributed to major advances in the field of TAAA repair [7–11]. However, open repair of TAAAs continues to be challenging and carries a substantial risk of perioperative morbidity and mortality, primarily due to the large extent of surgical trauma; hemorrhagic conditions; and ischemic insults involving the brain, spinal cord, kidneys, and viscera [12, 13]. Yet, to date, there has been a paucity of studies comprehensively analyzing the potential differential temporal impacts of baseline, anatomical, and procedural variables on the clinical outcomes of open repair of TAAAs, which led us to explore this issue from our institutional experiences.

Of note, in the present study, older age, high CPB time, and low surgical volume emerged as common risk factors for both early mortality and disabling complications, while several other factors (i.e., gender, hemoglobin level, and Crawford extent) showed modest associations with adverse outcomes. In addition to generally well-known risk factors, such as age and baseline hemoglobin level, in cardiovascular surgery [14], low surgical volume (< 20 cases) was found to be a significant independent predictor of mortality and disabling events in the present study. Notably, the impact of this surgical factor was stronger than that of both the Crawford extent and the urgency of surgery. The contribution of this surgical factor to outcomes may be associated with variables, such as differences in CPB strategy, needling skills, flexibility to deal with intraoperative events, and overall surgical flow, all of which are difficult to quantify for analyses. Studies in other fields, such as valve and coronary surgery, have also noted surgical volume as a predictor of operative outcomes, in addition to the effect of the learning curve; however, we believe the present study is unique as there is a lack of such data regarding open repair of TAAAs [15, 16]. Further studies are required to determine which subvariables within this surgical factor specifically increase operative risk, and the findings will be important, as such variables are likely to be correctable. Of note, CPB time was found to have its own independent impact on early outcomes even when the Crawford extent was considered in the multivariable model. It is a common expectation that a greater surgical extent, which accompanies a higher surgical risk, inevitably results in a longer CPB time (extent II or III vs. extent I, IV, or V); however, the present study revealed that CPB time along with surgical volume had a greater impact on early outcomes than the Crawford extent, which should be investigated further in a targeted study [17]. It is intuitive that CPB time may have a significant association with Crawford extent and surgical volume; however, the VIF test revealed that there was no significant multicollinearity among the risk variables, including CPB time, in the multivariable logistic regression models, demonstrating the independent association of CPB time with adverse outcomes.

It is plausible that the outcomes of any cardiovascular surgery may be affected by institutional and/or surgeon factors, as evidenced by large-scale data demonstrating the influence of institutional volume on mortality risks in coronary and valve procedures. Open surgery for TAAAs, which is one of the most extensive and challenging procedures in the field of cardiovascular surgery, is likely to be more affected by such factors; however, the evaluation of this issue has been hindered by a lack of real-world data to compare outcomes. In this regard, the present study found that surgical volume was significantly associated with the risks of mortality and disabling complications, with low surgical volume being associated with significantly worse outcomes. In this surgical factor, a number of variables might have been involved, including different CPB strategies, suturing techniques, overall cumulative experience, and capacity of handling unexpected intraoperative events; however, such individual components could not be analyzed separately in this limited observational study. Nevertheless, the association of surgical volume with outcomes demonstrated in the present study suggests that referring surgical candidates with TAAAs to high-volume centers may be a reasonable option, and this needs explorative studies for further validation.

Extending the analyses to the late follow-up period also yielded interesting findings of varying temporal impacts among significant risk variables. For instance, age, hemoglobin level, and renal function showed significant or modestly significant impacts on long-term mortality throughout the follow-up period (stable proportional hazards function). Meanwhile, greater Crawford extent (i.e., extent II), low surgical volume, and high CPB time had significant impacts in the early phase, which were neutralized over the late follow-up phase. These findings seem intuitive in that uncorrectable factors (age, hemoglobin level, and renal function) have sustained long-term influences, while surgery-related factors (surgical volume and CPB time) and corrected factors (Crawford extent) lose their impacts in the late phase. Further ROC analyses on continuous risk variables generally revealed that the best cutoff values differentiating mortality risk lie near normal reference values (hemoglobin level of 11.6 g/dL and GFR of 66 mL/min/1.73 m^2^), indicating the already hazardous impacts of such factors, even at lower normal limits.

We believe the findings should be validated with a larger dataset because the number of cases involving spinal cord deficit (SCD) was rather small (*n* = 32) to perform robust multivariable modeling. Considering that sex is an important factor in cardiovascular surgery, further studies focusing on this factor in terms of TAAAs will be interesting to the cardiovascular surgical community.

### Limitations

An important limitation of this study is its single-center, retrospective, observational design. The study data were collected from medical records spanning a 28-year period, with significant variations in surgeons, CPB strategies, and surgical volumes during the study period. Thus, heterogeneity and residual confounding variables might have affected the results of our study, despite the use of statistical modeling. Moreover, the numbers of patients experiencing individual components of major adverse events (SCD in particular) were too small to perform robust multivariable modeling to determine the independent risk factors for each outcome. Finally, as our data relied on the experience from a single tertiary referral center, the results of the study may have limited generalizability to other settings. The absence of fenestrated endografts at our institution is a limitation in our practice. Many centers adopting endovascular fenestrated endografts have reported a decline in open surgical volume, particularly for open extent II procedures where a hybrid approach is feasible. This shift in procedural preferences may have significant implications for surgical outcomes in the future as the volume of open surgeries continues to decrease.

## Conclusions

Open repair of TAAAs carries significant risks of early mortality and disabling complications. There were differential temporal impacts of perioperative risk variables on mortality in open repair of TAAAs, with older age and low hemoglobin level having significant impacts throughout the postoperative period, and low surgical volume, high CPB time, and Crawford extent II having impacts in the early postoperative phase.

### Electronic supplementary material

Below is the link to the electronic supplementary material.

Supplementary Material 1

Supplementary Material 2

Supplementary Material 3

Supplementary Material 4

Supplementary Material 5

Supplementary Material 6

## Data Availability

The datasets generated and analyzed during the current study are available from the corresponding author on reasonable request.

## Abbreviations

TAAA: Thoracoabdominal aorta aneurysm
CPB: Cardiopulmonary bypass
LOCS: Low cardiac output syndrome
VIF: Variance inflation factor
AUC: Area under the curve
HR: Hazard ratio
TCA: Total circulatory arrest
OR: Odds ratio
CI: Confidence interval
SCD: Spinal cord deficit
ARF: Acute renal failure
BMI: Body mass index
Hb: Hemoglobin
COPD: Chronic obstructive pulmonary disease
TEVAR: Thoracic endovascular aneurysm repair
PCI: Percutaneous coronary intervention
CABG: Coronary artery bypass grafting
CVA: Cerebrovascular accident
TIA: Transient ischemic attack
ESRD: End-stage renal disease
GFR: Glomerular filtration rate
LSCA: Left subclavian artery
ICA: Intercostal artery
LA: Lumbar artery
