# Supplementary material for: Differential impacts of clinical, anatomical, and procedural factors on early and late mortality following open thoracoabdominal aortic repair: a retrospective observational study
Source: J Cardiothorac Surg. 2024 Jun 24;19:360. doi: 10.1186/s13019-024-02933-2 (PMC11194940; doi:10.1186/s13019-024-02933-2)
Supplement: Supplementary file 1 — Supplementary Material 1 [file 13019_2024_2933_MOESM1_ESM.docx]

**Supplementary Table 1. Outcomes of high-volume surgeons (operator 1, 2, 3) before and after first 20 cases.**

|  | Case volume<20 | Case volume≥20 | *P* value |
| --- | --- | --- | --- |
|  | N=57 | N=193 |  |
| Operator |  |  | 0.68 |
| 1 | 19 (33.3) | 72 (37.3) |  |
| 2 | 19 (33.3) | 53 (27.5) |  |
| 3 | 19 (33.3) | 68 (35.2) |  |
| Early mortality, n (%) | 13 (22.8) | 19 (9.8) | 0.019 |
| Disabling complications, n (%) | 18 (31.6) | 43 (22.3) | 0.21 |
| The composite events, n (%) | 18 (31.6) | 43 (22.3) | 0.21 |
