# Supplementary figures and images for: Differential impacts of clinical, anatomical, and procedural factors on early and late mortality following open thoracoabdominal aortic repair: a retrospective observational study
Source: J Cardiothorac Surg. 2024 Jun 24;19:360. doi: 10.1186/s13019-024-02933-2 (PMC11194940; doi:10.1186/s13019-024-02933-2)

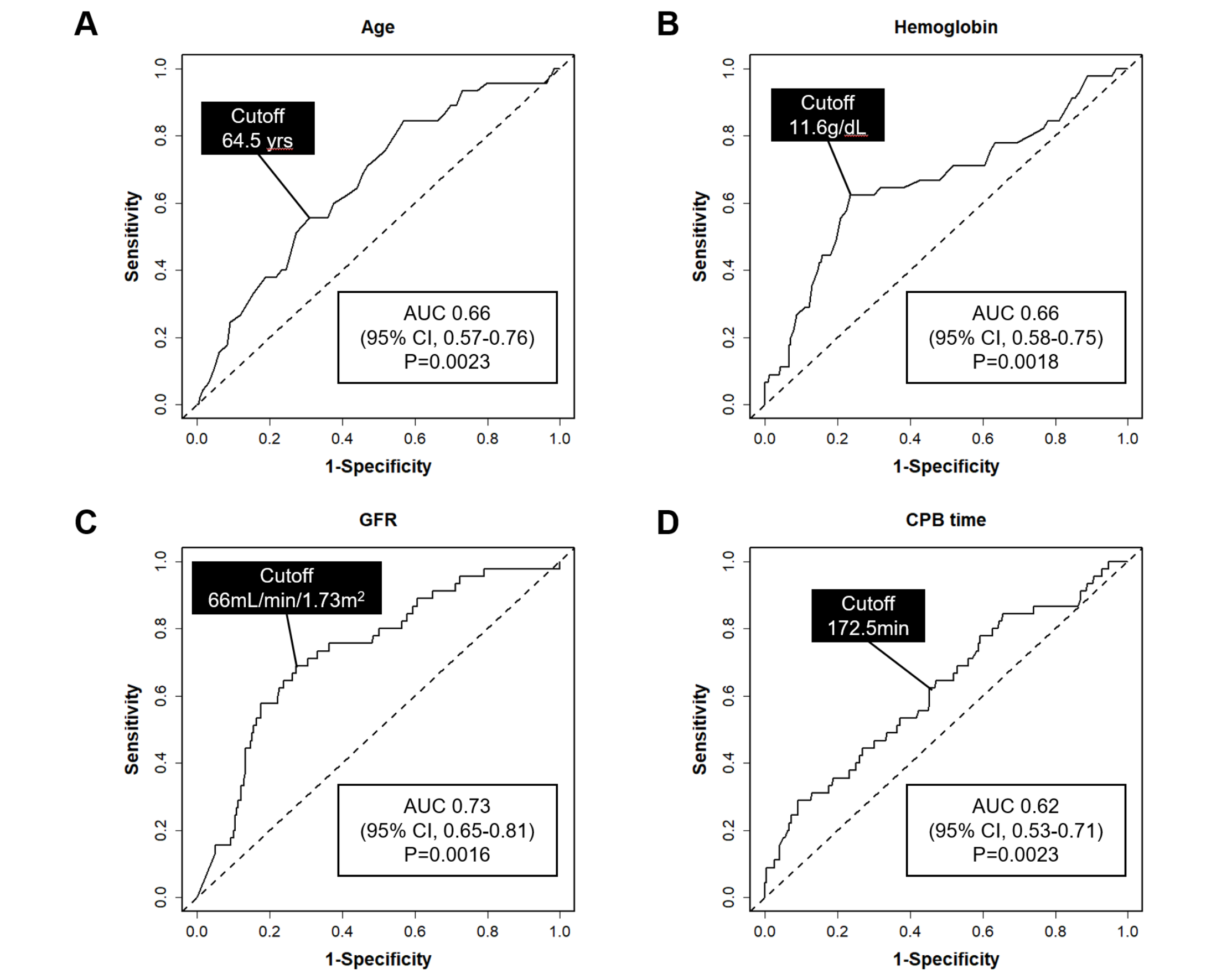

Supplement: Supplementary file 2 — Supplementary Material 2 [file 13019_2024_2933_MOESM2_ESM.png]

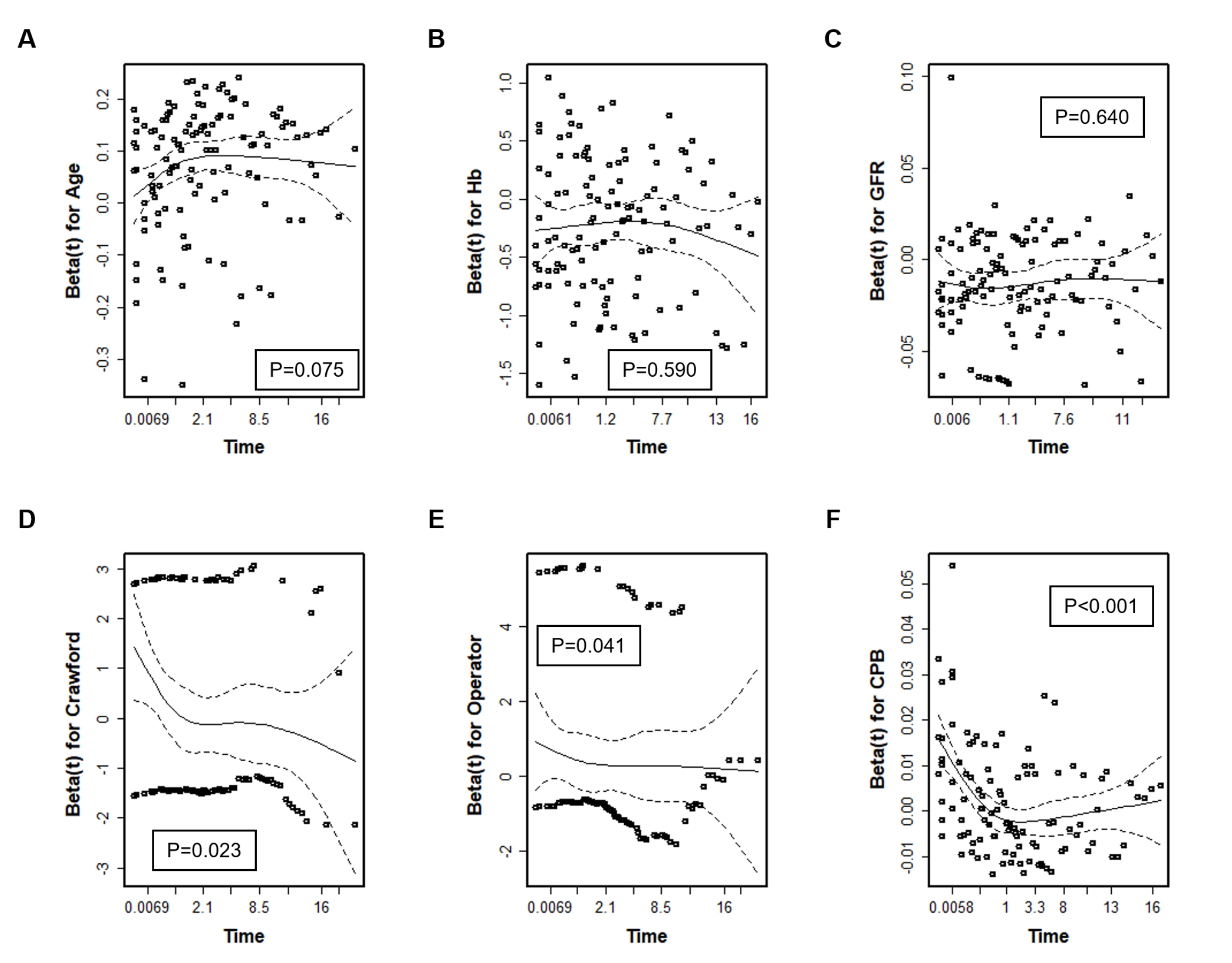

Supplement: Supplementary file 3 — Supplementary Material 3 [file 13019_2024_2933_MOESM3_ESM.png]

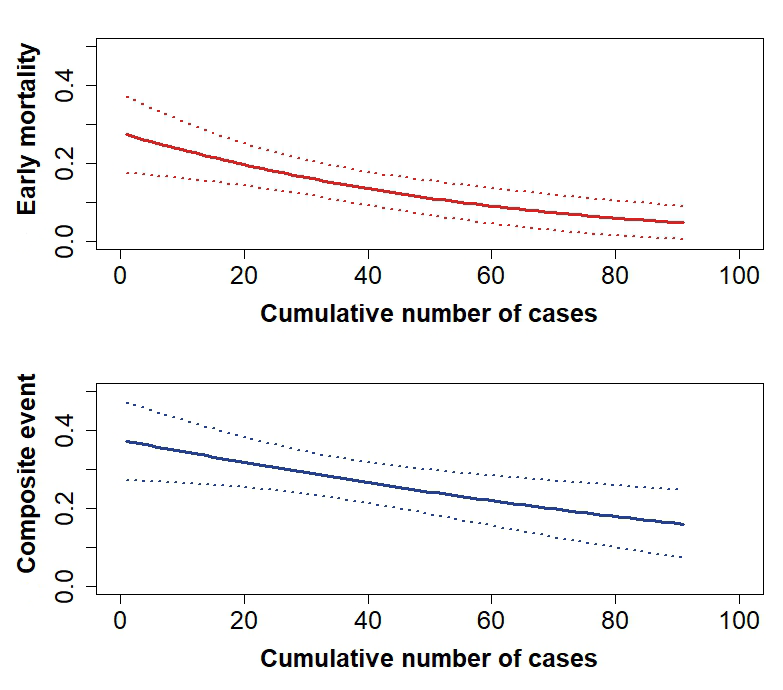

Supplement: Supplementary file 4 — Supplementary Material 4 [file 13019_2024_2933_MOESM4_ESM.png]

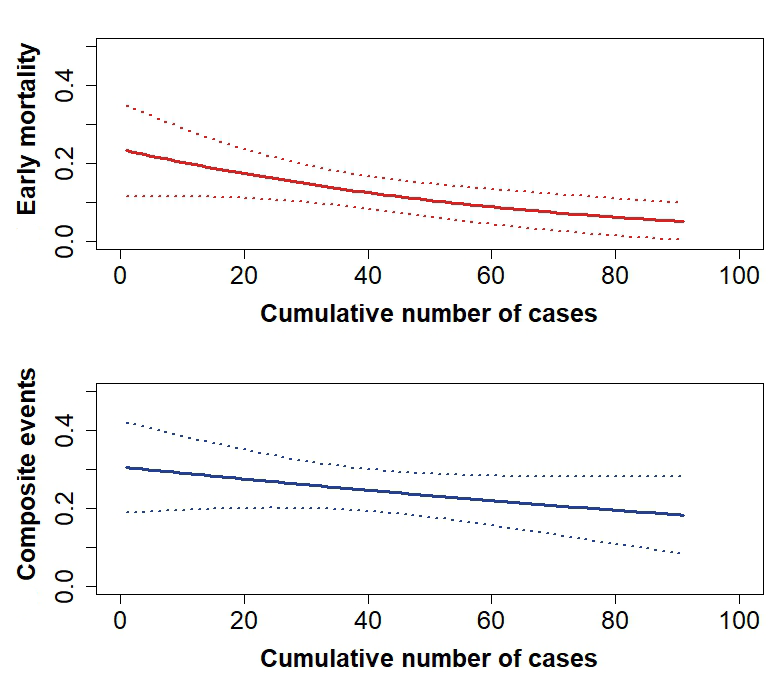

Supplement: Supplementary file 5 — Supplementary Material 5 [file 13019_2024_2933_MOESM5_ESM.png]
