# Supplementary material for: Differential impacts of clinical, anatomical, and procedural factors on early and late mortality following open thoracoabdominal aortic repair: a retrospective observational study
Source: J Cardiothorac Surg. 2024 Jun 24;19:360. doi: 10.1186/s13019-024-02933-2 (PMC11194940; doi:10.1186/s13019-024-02933-2)
Supplement: Supplementary file 6 — Supplementary Material 6 [file 13019_2024_2933_MOESM6_ESM.docx]

**Supplementary Figure 1.** Receiver operating characteristic curves for age, hemoglobin, glomerular filtration rate, and CPB time regarding early mortality. CPB: cardiopulmonary bypass

**Supplementary Figure 2.** Violation of Cox proportional hazards assumption tested by the Schoenfeld residual. (a) Age; (b) Hb; (c) GFR; (d) Crawford extent; (e) surgical volume; and (f) CPB. Hb: hemoglobin; GFR: glomerular filtration rate; CPB: cardiopulmonary bypass

**Supplementary Figure 3**. Generalized linear regression model. Cumulative number of surgical cases and outcomes at the individual surgeon level, including all surgeons. *P*=0.001 for mortality and *P*=0.015 for the composite events.

**Supplementary Figure 4.** Generalized linear regression model. Cumulative number of surgical cases and outcomes at the individual surgeon level, including high-volume surgeons. *P*=0.016 for mortality and *P*=0.21 for the composite events.

**Supplementary Table 1.** Outcomes of high-volume surgeons (operator 1, 2, 3) before and after first 20 cases.
